# Adaptation changes in dynamic postural control and contingent negative variation during backward disturbance by transient floor translation in the elderly

**DOI:** 10.1186/1880-6805-31-12

**Published:** 2012-05-24

**Authors:** Katsuo Fujiwara, Maki Maekawa, Naoe Kiyota, Chie Yaguchi

**Affiliations:** 1 Department of Human Movement and Health, Graduate School of Medical Science, Kanazawa University, 13-1 Takara-machi, Kanazawa, 920-8640 Japan; 2 Department of Rehabilitation Science, Osaka Health Science University, 1-9-27 Temma, Kita-ku, Osaka, 530-0043, Japan; 3 Department of Physical Therapy, Faculty of Human Science, Hokkaido Bunkyo University, 5-196-1, Kogane-chuo, Eniwa, 061-1449, Japan

**Keywords:** Postural disturbance, Floor translation, Adaptation, Contingent negative variation, Elderly subject, Anticipatory postural control

## Abstract

**Background:**

We investigated adaptation changes in dynamic postural control and contingent negative variation (CNV) in 13 young and 12 elderly adults. Subjects repeatedly underwent backward postural disturbance by a forward floor translation (S2) 2 s after an auditory warning signal (S1). Initial and second sets were conducted, each set with 20 trials. Posterior peak position of the center of pressure in the anteroposterior direction (CoPy) after S2 was identified. Electroencephalograms from Cz were averaged for each set, and the CNV negative peak was identified.

**Results:**

Compared with the first trial, the posterior peak position of CoPy changed significantly forward from the 12th trial in the young and from the 19th trial in the elderly during the initial set. The mean of the posterior peak position was more forward in second set than in the initial set for both groups and was significantly backward in the elderly compared to the young for both sets. These findings indicate that subjects in both groups adapted better to the postural disturbance in the second set than in the initial set, and the adaptation was later in the elderly. Late CNV in the young started to increase negatively from the middle of the S1-S2 period and peaked just before S2. Peak CNV amplitude was larger in the second set than in the initial set. In contrast, late CNV in the elderly exhibited no negative increase as in the young and peaked in the middle of the S1-S2 period, which was followed by gradual decreasing toward S2. No adaptive changes were found in late CNV for the elderly.

**Conclusions:**

It is conceivable that reduced activation of the frontal lobe may be one of the factors contributing to the decrease in postural adaptability in the elderly. The elderly may use various brain regions for the adaptation of dynamic postural control compared with the young.

## Background

Deterioration of equilibrium function is considered to be a primary cause of falls among the elderly. Falls often occur in a dynamic situation, such as when perturbation is externally applied and during walking. Falls among the elderly cause serious problems, such as prolonged immobility due to head injury or hip fracture, and the increased cost for health and social services due to the immobility [1,2]. Backward falls pose a greater risk for serious injuries [3]; thus, it is important to develop equilibrium training to prevent backward falls in the elderly. In equilibrium training, it is critical to target the improvement of postural control under dynamic conditions, such as forward floor translation, when the entire body leans backward, and to simultaneously evaluate the adaptability of the dynamic postural control. It is well known that anticipation of disturbance timings and preparation for the disturbance play important roles in dynamic postural control. These functions are carried out in the frontal lobe, including the prefrontal cortex, supplementary motor area, premotor area, and primary motor area [4,5]. However, experimental studies have not addressed the role of frontal lobe function in the adaptability of dynamic postural control.

In order to investigate the adaptability of dynamic postural control, it is appropriate to use repeated forward floor translation. Forward floor translation simulates the slip, which often results in falls [6-8], in which displacement of the center of pressure in the anteroposterior direction (CoPy) is an index of the magnitude of backward postural disturbance. We have previously proposed a procedure for setting the amplitude and speed of forward floor translation for backward balance training, based on the CoPy position during an extreme backward leaning (EBL) posture, which is considered the posterior limit of stability [9]. Using such evaluation and perturbation methods, we will be able to investigate the individual adaptability of the dynamic postural control in response to backward disturbance for different subjects.

Contingent negative variation (CNV) is the negative slow potential obtained by averaging electroencephalograms (EEGs) between warning (S1) and response (S2) signals [10]. Presentation of S1 before the floor translation as S2 will allow an evaluation of the preparation state and the attention directed to the translation by measurement of the CNV [11,12]. It has been suggested that various cortical and subcortical regions, such as frontal cortical areas, somatosensory cortex and basal ganglia, are involved in the production of the late component of CNV (late CNV) [13]. Late CNV is reported to reflect the motor preparation process and anticipatory attention directed to S2 [14,15], and its amplitude increases with larger amounts of attention directed to S2 [15]. In addition, it was suggested that CNV peak corresponds to a peak of anticipatory attention and/or onset of attentional shift to the objects other than S2, such as sensory information and output of motor command [11,12,16]. Previous studies with backward floor translation and arm abduction task while standing have indicated that changes in the late CNV amplitude and CNV peak correlated to muscle activity and CoPy before and after postural disturbance [12,17]. On the other hand, for the elderly performing finger flexion, late CNV was smaller in the younger elderly than in the young, but such negative potential was not observed in the older elderly [13]. This finding suggests a decrease in attention and motor preparation for S2 and/or deterioration of brain regions related to the origin of CNV (mainly frontal lobe). In the present study, using floor translation as S2 while standing, we might expect that negative deflection of late CNV would be smaller in the elderly than in the young. In addition, by comparing CNV between initial and second sets of repeated floor translations and demonstrating relationships between the adaptive changes of CNV, muscle activity, and CoPy, we will be able to investigate the functional role of the frontal lobe in postural adaptation.

In this study, we repeatedly applied forward floor translation as S2. Disturbance intensity caused by translation was equal for all young and elderly subjects, and adaptation changes in postural control and CNV in response to the backward postural disturbance were investigated. Working hypotheses were as follows: (1) With the repeated floor translation, backward displacement of CoPy would be smaller as a result of adaptive improvement of dynamic postural control. The adaptive improvement would be later in the elderly than in the young subjects.(2) with the postural adaptation, late CNV amplitude would increase in the young subjects, but in the elderly subjects, the CNV change would not be recognized.

## Methods

### Subjects

Subjects were 13 healthy young adults (7 men, 6 women) and 12 healthy elderly adults (6 men, 6 women). Mean age, height, weight, and foot length (FL) were 22.2 years [standard deviation (SD) = 4.8], 168.8 cm (SD = 7.3), 60.5 kg (SD = 6.8), and 25.0 cm (SD = 1.6) in young subjects and 65.5 years (SD = 3.6), 157.8 cm (SD = 6.9), 62.4 kg (SD = 5.8), and 23.7 cm (SD = 1.0) in elderly subjects, respectively. No subject had any history of neurological or orthopedic impairment. All young subjects had belonged to ball-game clubs, such as basketball and soccer, for more than 6 consecutive years. Elderly subjects, who could perform activities of daily living without assistance, were recruited from the suburban areas of Kanazawa and Suzu city of Japan. Many of them were relatively healthy people who performed daily work, such as farming, or participated in exercise programs several times a week. Informed consent was obtained from all subjects in accordance with the Declaration of Helsinki following an explanation of our experimental protocols, which were approved by the Institutional Ethics Committee of Kanazawa University.

### Apparatus and data recording

A platform (FPA34; Electro-design, Japan) was used to measure CoPy. The CoPy electronic signals were sent simultaneously to one computer (PC9801BX2; NEC, Japan) to determine the CoPy position and to another computer for analysis. The former received CoPy data via an A/D converter (PIO9045; I/O-Data, Japan) at 20 Hz with 12-bit resolution and could generate a buzzer sound when the CoPy was located within ±1 cm of the required position. The CoPy position was calculated and shown as the percentage distance from the heel in relation to FL (%FL). The force platform was fixed to a handmade table that was movable horizontally in an anteroposterior direction by a computer-controlled electric motor (VLA-ST-60-60-0300; THK, Japan). Direction, velocity, and amplitude of platform movement were adjusted using Cutey Wave II software (Sanmei-Denshi, Japan). S1 was an auditory stimulus delivered via earphones with frequency, intensity, and duration of 2,000 Hz, 35 dB above the threshold, and 50 ms, respectively. S2 was a transient forward floor translation. Onset of translation was detected by an accelerometer (AG-2 GB; Kyowa, Japan) fixed to the force platform.

Ag-AgCl cup electrodes (diameter, 8 mm) for recording EEG were affixed to the scalp at Fz, Cz, and Pz in accordance with the international 10–20 system, and referenced to linked ear lobes. A ground electrode was placed at Fpz. Electrooculography (EOG) was recorded from a pair of electrodes placed above and below the left eye. To fix eye position, subjects were instructed to gaze at a fixation point presented on an Eye-trek face-mounted display (FMD011F; Olympus, Japan). Surface electrodes (P-00-S; Ambu, Denmark) were used in bipolar derivation to record surface electromyography (EMG) of the following muscles on the left side: the rectus abdominis (RA) erector spinae (ES), rectus femoris (RF), biceps femoris (BF), tibialis anterior (TA), medial head of the gastrocnemius (GcM), and soleus (Sol). For each muscle, electrodes were fixed after shaving and cleaning the skin with alcohol. The electrodes were aligned along the long axis of the muscle with an inter-electrode distance of about 3 cm. Electrode input impedance was < 5 kΩ. Signals from electrodes were amplified (EEG, ×40,000; EOG, ×4,000; EMG, ×4,000) and band-pass filtered (EEG, 0.05–100 Hz; EOG, 0.05–30 Hz; EMG, 5–500 Hz) using an amplifier (Biotop 6R12; NEC-Sanei Japan).

For subsequent analyses, all electrical signals were sent to a computer (Dimension E521; Dell, Japan) via an A/D converter (ADA16-32/2(CB) F; Contec, Japan) at 1 kHz with 16-bit resolution.

### Procedure

All measurements were carried out while subjects were standing barefoot, with feet 10 cm apart and parallel on the force platform, and upper limbs crossed in front of the chest. To prevent falls due to floor translation, subjects wore a harness around the chest.

First, the mean positions of CoPy were measured while subjects maintained a quiet standing (QS) posture for 10 s. The mean value of the five trials was adopted as the QS position. Next, the mean CoPy position during EBL posture was measured twice. Subjects gradually leaned backward from QS for approximately 5 s, pivoting at the ankles with the rest of the body kept aligned, and then maintained this EBL posture for 3 s. The more posterior CoPy mean position of two trials was adopted as the EBL position, and the posterior peak position of the CoPy in the adapted trial was defined as the EBL peak position.

**Figure 1:**
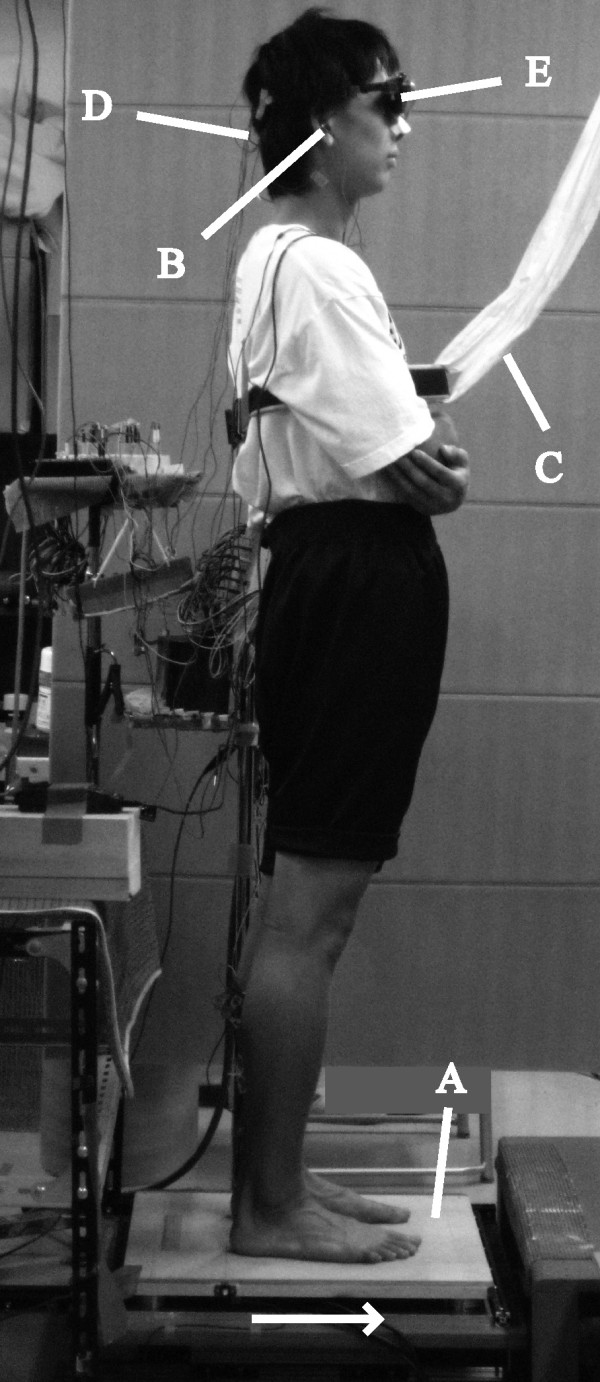
**Experimental setup. A: platform, B: earphone, C: harness, D: Ag-AgCl cup electrodes, E: Eye-trek face-mounted display.** Direction of platform movement is indicated by the *arrow*.

Next, the intensity of floor translation (amplitude and velocity) was set for each subject, and then the experimental session was carried out (Figure 1). During both the setting of the intensity and the experiment, subjects maintained the CoPy position within the QS position ± 1 cm, which was presented by a buzzer sound for at least 3 s, until S2 onset. S1 was randomly presented in 1–2 s after the experimenter stopped the buzzer sound, and then S2 started 2 s after S1. Subjects were instructed to avoid changing of the initial foot position or falling in response to S2.

The intensity of floor translation was set according to the following two processes [9]. First, the translation velocity was determined as 25, 20, 15, or 10 cm/s. To begin, a floor translation of 20 cm/s was applied at 5- and 10-cm amplitudes. If the posterior peak of CoPy after the translation in either of these amplitudes was located between the EBL and EBL peak positions, 20 cm/s was adopted as the translation velocity. If not, the velocity was reduced or increased until the posterior peak of CoPy in either of these amplitudes was located between those positions. Second, the translation amplitude was determined. A linear regression line was drawn through the two coordinates of the floor translation amplitude and the posterior peak of CoPy at the velocity determined in step 1. Based on the line, the translation amplitude, at which the posterior peak would be located midway between the EBL and EBL peak positions was determined. The mean ± SD of the adopted translation velocity and amplitude was 20.4 ± 2.5 cm/s and 8.6 ± 2.1 cm in the young, and 12.9 ± 3.3 cm/s and 5.6 ± 1.5 cm in the elderly, respectively.

In the experimental session, two sets of trials (initial and second sets) were conducted, each set with at least 20 trials. Trials were excluded if foot position changes were observed or if deviation of CoPy over ± 1 cm from the QS position was noted before S2. A 30-s standing rest and a 3-min seated rest were taken between trials and sets, respectively.

### Data analysis

All data analysis was performed using BIMUTAS II software (Kissei Comtec, Japan). To evaluate the magnitude of backward disturbance in response to the floor translation, the posterior peak of CoPy after S2 was identified in each trial, and the distance from EBL position to this peak position was defined as the displaced position of CoPy. If more than 20 trials were conducted within a set, the mean value of the displaced positions in the trials after the 20th was defined as the value in the 20th trial. Displaced positions from the 16th to 20th trials in the second set were averaged, and the value was defined as the position in the final phase. Moreover, all trials in each set were averaged to investigate the change in displaced position of the CoPy corresponding to the change in CNV. To check whether the posterior peak was between the EBL and EBL peak positions, the difference between these positions was calculated and compared with the displaced position.

EEG, EMG, and CoPy waveforms from 300 ms before S1 to 3,000 ms after S2 were averaged for each set. The mean amplitude for the 300-ms period before S1 was used as a baseline of averaging. Preceding EMG averaging, all EMGs were 40-Hz high-pass-filtered to exclude electrocardiographic and movement artifacts, and then full-wave-rectified (rEMG). Trials with eye blinks or movement artifacts (voltage at EOG or any EEG electrode exceeding ± 100 μV) between 300 ms before S1 and S2 were excluded from the averaging, and at least 12 trials were adopted for each set. In each averaged waveform, mean amplitudes for every 100-ms period were calculated. Waveforms recorded from Cz, in which late CNV was the maximum in both sets, were used for CNV analyses. The mean amplitude of the 100-ms period just before S2 was defined as the late CNV amplitude.

In order to investigate the relationship between the CNV peak and EMG activity before S2, averaged waveforms of EEG and rEMG were 4-Hz low-pass-filtered [12]. The EEG waveform exceeded baseline negatively in about 300 and 500 ms after S1 in the young and elderly, respectively. Therefore, the maximal negative potential from 500 ms after S1 to S2 was defined as the CNV peak, and its latency relative to S2 and amplitude from baseline were calculated (CNV peak latency and amplitude). Around the CNV peak, a continuous increase of TA background activity started, especially in the young. Some elderly subjects showed a transient increase of TA around the peak. Accordingly, the muscle increasing timing was identified as the point in which the amplitude first exceeded the mean amplitude of the S1-S2 period for >100 ms from around the CNV peak to S2, and the start time relative to S2 was calculated. In 4 of 26 cases, combining the initial and second sets for the young, and in 6 of 24 cases for the elderly, the increases of background activity were observed in other anterior muscles (RA: 8 cases; RF: 2 cases), and thus these muscles were analyzed.

**Figure 2:**
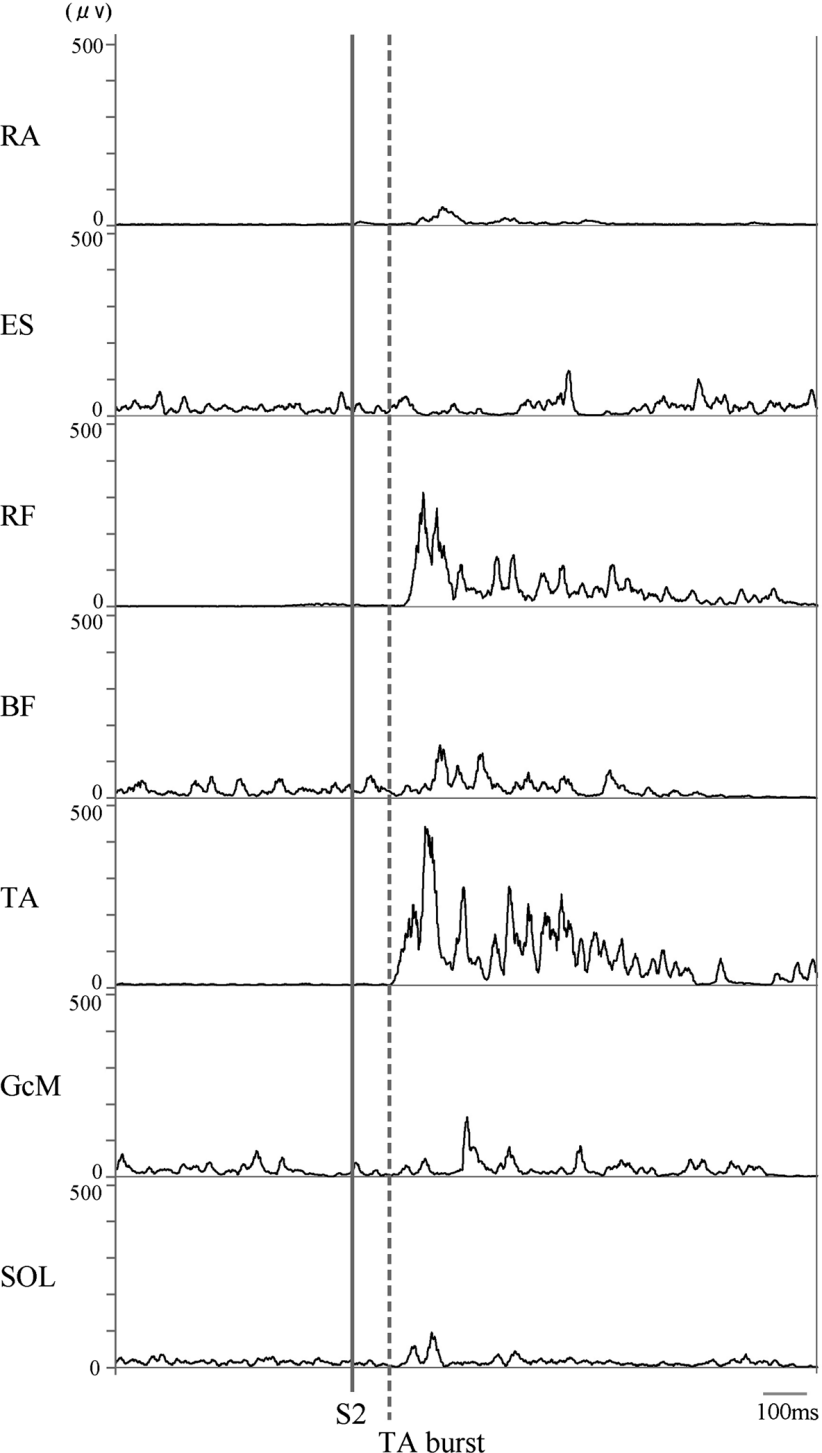
**Representative waveforms of EMG burst activity in response to S2 for one subject.** Solid and dashed lines indicate onsets of S2 and TA burst activity.

Observing rEMG waveforms after S2 (Figure 2), the earliest burst activation was found in TA. Therefore, the following analyses were conducted for TA after S2 in the trials adopted for averaging. In each trial, the envelope line of the TA burst continuing at least 50 ms was identified by visual inspection of the EMG trace on a computer. The time point at which the TA burst deviated more than the mean +2 SD from the background activity of the standing posture before S1 was defined as the burst onset of TA, and the onset time from S2 was measured. To analyze the burst activity level, rEMG waveforms of TA in the period from −500 to +1,000 ms with respect to the burst onset were then averaged for each set. The averaged waveforms were smoothed using a 40-Hz low-pass filter, and then a peak was identified. Peak amplitude from the baseline and peak time with respect to burst onset were measured. Peak amplitude was normalized by the EMG mean amplitude for 3 s during EBL posture.

### Statistical analysis

Shapiro-Wilks and Levene’s tests confirmed that all data satisfied the assumptions of normality and equal variance, respectively. To evaluate group differences in CoPy parameters, and CNV peak and late CNV amplitudes, Student’s t-test was used. To compare QS and EBL positions in this study with those in previous studies, and to investigate whether EMG activity before S2 was significantly increased relative to baseline, the one-sample *t*-test was used. A two-way analysis of variance (ANOVA) was used to assess the effects of trial and group (40 trials × 2 groups) on the displaced position of CoPy. When a significant main effect of trial was shown, the differences between the first trial and the other trials by group were evaluated using Dunnett’s test to further assess the adaptability in response to the floor translation. Two-way ANOVA was also performed to assess the effects of set and group (2 sets × 2 groups) on mean displaced position among 20 trials, CNV peak latency, start time of TA increase before S2, and onset time, peak amplitude, and peak time of TA burst. In order to evaluate the magnitude of correlation among adaptive changes of measurement parameters, differences between initial and second sets were calculated, and Pearson’s correlation was used between the differences in each parameter. The alpha level for all tests was set at *p* < 0.05. All statistical analyses were performed using SPSS 14.0 J software (SPSS Japan, Japan)

## Results

The mean ± SD of the QS position was 45.3 ± 4.9%FL in the young and 42.1 ± 3.7%FL in the elderly, with no significant group difference. EBL position, EBL peak position, and distance from EBL to EBL peak positions were 18.6 ± 2.4%FL, 13.5 ± 2.9%FL, and −5.1 ± 1.6%FL in the young, and 21.8 ± 4.6%FL, 15.9 ± 3.9%FL, and −5.9 ± 1.4%FL in the elderly, respectively. EBL position in the elderly was significantly forward compared to the young (*t*_*12*_ = 2.16; *p* < 0.05). There were no significant group differences in the other measurements. Compared with previously reported CoPy, only EBL position in the elderly was significantly backward (25.5%FL) [18] (*t*_*12*_ = 2.78, *p* < 0.05).

**Figure 3:**
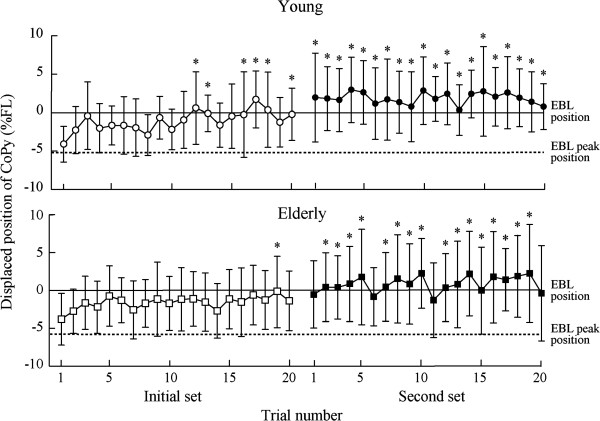
**Means and standard deviations of displaced position of CoPy in response to the floor translation in each trial.***Upper*: young subjects; *lower*: elderly subjects. *Asterisk* indicates a significant difference in a trial compared to the first trial in the early set (*p* < 0.05).

**Figure 4:**
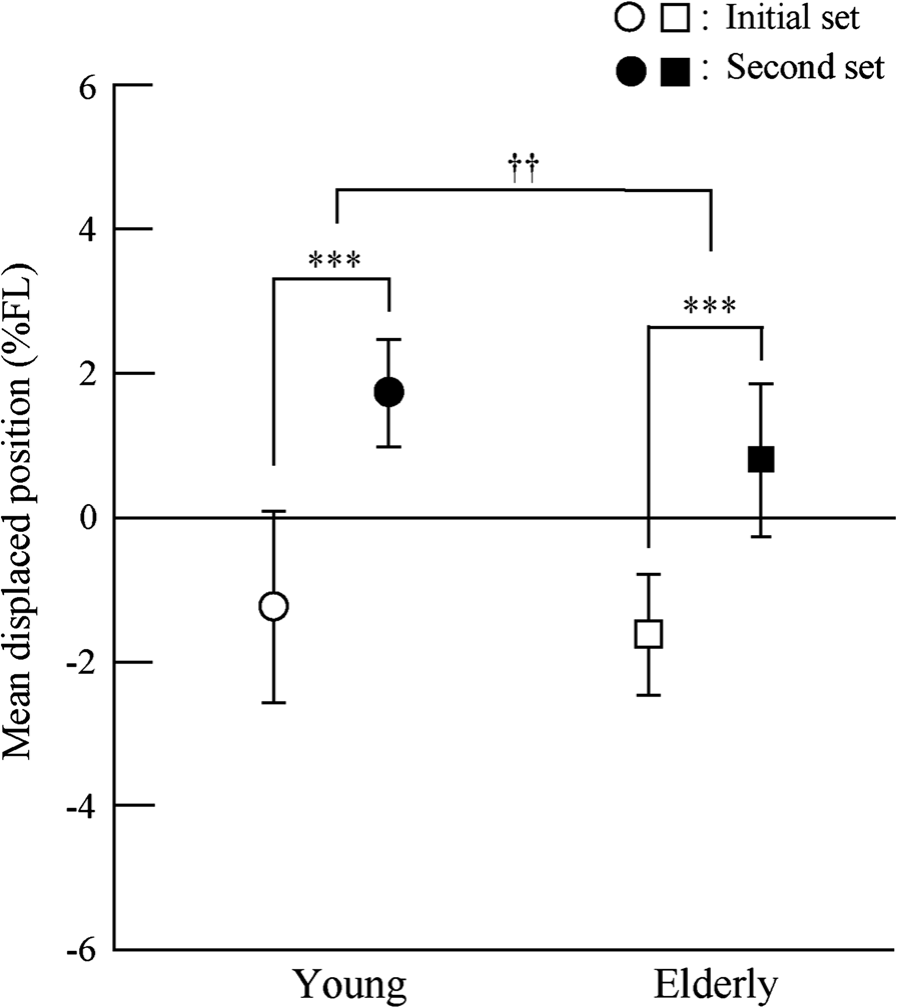
**Means and standard deviations of mean displaced position among 20 trials in each set.** Asterisk and dagger indicates a significant effect of set and group, respectively (**, ††: *p* < 0.01; ***: *p* < 0.001).

Figure 3 shows the displaced position of the CoPy in response to the floor translation in each trial. In the first trial of the initial set, the mean ± SD of the displaced position was −4.2 ± 2.3 %FL in the young and −3.8 ± 3.4 %FL in the elderly, with no significant group difference. A significant effect of trial (*F*_*39, 897*_ = 7.32, *p* < 0.001) on the displaced position was found, but there were no significant group effects or interactions between trial and group. The displaced position after the 12th and 19th trials of the initial set for the young and elderly, respectively, was significantly more forward than in the first trial of the initial set (*p* < 0.05). Figure 4 shows the mean displaced position among 20 trials in each set. Significant effects of set (*F*_*1, 38*_ = 151.29, *p* < 0.001) and group (*F*_*1, 38*_ = 7.74, *p* < 0.01) were found, but there was no significant interaction between them. The mean displaced position in the second set was significantly forward compared to the initial set, and in the elderly it was significantly backward compared with the young. However, displaced positions in the final phase were 1.6 ± 3.4 %FL in the young and 1.4 ± 5.1%FL in the elderly, with no significant group difference.

**Figure 5:**
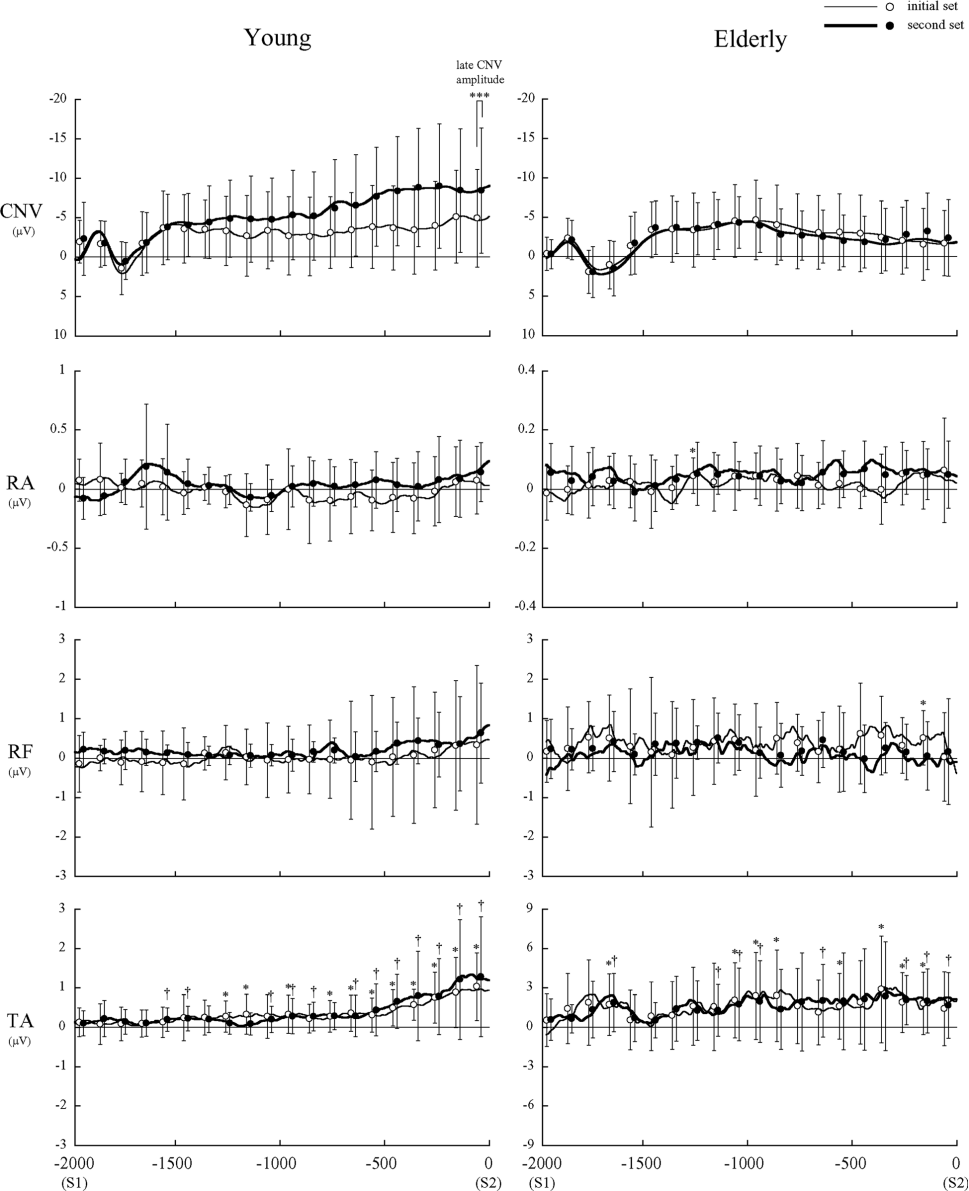
**Grand average waveforms of CNV and EMG between S1 and S2.** Means and standard deviations of mean amplitude for every 100-ms period were overlapped on the waveforms. *Thin line*: initial set; *thick line*: second set. The * and † indicate a significant increase from baseline in the initial set and second set, respectively. ***Indicates a significant difference in late CNV amplitude between the initial and second sets in the young.

Grand average waveforms of CNV and EMG between S1 and S2, overlapping means, and the SD of mean amplitude for every 100-ms period are shown in Figure 5. In the young, late CNV started to increase negatively from the middle of the S1-S2 period and peaked just before S2 (CNV peak latency, initial set: −166 ± 154.4 ms; second set: −203 ± 191.8 ms). In contrast, late CNV in the elderly exhibited no negative increase as in the young and peaked at the middle of the S1-S2 period, which was followed by gradual decreasing toward S2 (CNV peak latency, initial set: −800 ± 294.7 ms; second set: −689 ± 433.7 ms). Only three elderly subjects showed clear negative increases of late CNV in the second set. CNV peak latency was significantly shorter in the young than in the elderly (main effect of group: *F*_*1,23*_ = 110.10, *p* < 0.001). Only in the young, CNV peak amplitude (*t*_*12*_ = 2.25, *p* < 0.05) and late CNV amplitude (*t*_*12*_ = 2.23, *p* < 0.05) in the second set were significantly larger than in the initial set.

**Figure 6:**
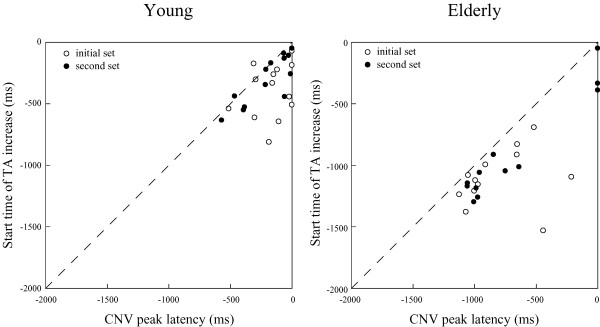
**Relationships between CNV peak latency and start time of TA increase.** In the second set, significant correlations were found for both groups (young: *r* = 0.83; elderly: *r* = 0.96).

TA background activity gradually increased from around the CNV peak point toward S2 for the young (start time of TA increase, initial set: −394 ± 219.2 ms; second set: −305 ± 196.9 ms). For the elderly, the TA increase started around the CNV peak point (start time of TA increase, initial set: −1,104 ± 230.3 ms; second set: −906 ± 411.9 ms). The start time of the TA increase was significantly shorter in the young than in the elderly (main effect of group: *F*_*1, 23*_ = 85.53, *p* < 0.001). In most subjects, the start timing preceded the CNV peak timing, and in the second set, high positive correlations were found between the start time of TA increase and CNV peak latency in the young (*r* = 0.83) and elderly (*r* = 0.96) (Figure 6). In 5 of all 26 cases for the young, and in 6 of all 24 cases for the elderly, the increases of background activity were followed by a forward shift of the CoPy.

For the TA burst in response to S2, the onset time was significantly later in the elderly (initial set: 105 ± 7.6 ms; second set: 107 ± 9.3 ms) than in the young (initial set: 93 ± 7.2 ms; second set: 92 ± 7.9 ms) (main effect of group: *F*_*1,23*_ = 20.79, *p* < 0.05). Peak amplitude was significantly smaller in the elderly than in the young (main effects of group: *F*_*1,23*_ = 10.92, *p* < 0.01). Peak time was significantly later in the elderly (initial set: 84 ± 41.0 ms; second set: 75 ± 33.5 ms) than in the young (initial set: 64 ± 24.1 ms; second set: 41 ± 15.1 ms), but shortened in the second set compared with the initial set (main effects of group: *F*_*1,23*_ = 7.65, *p* < 0.05; set: *F*_*1,23*_ = 5.95, *p* < 0.05). Only in the elderly, for the difference between sets, the onset time of TA burst was negatively correlated with CNV peak latency (*r* = −0.71) and start time of TA increase (*r* = −0.62).

## Discussion

EBL position is an index of the posterior limit of stability [19]. From our previous studies, the EBL position in young adults was 19%FL and in the elderly was 25.5%FL [18]. In the present study, the EBL position in the young adults (18.6%FL) was not significantly different from the value previously reported, but in the elderly (21.8%FL) was significantly backward. Therefore, elderly subjects in this study would be categorized as having relatively high stability.

In the first trial of the floor translation task, the posterior peak position of the CoPy was displaced between the EBL position and EBL peak positions in both groups. No significant group difference was found in the distance from the EBL position to the peak position, which was referred to as the displaced position of the CoPy. These results indicate that postural disturbance could be applied to young adults and elderly with a relatively equal intensity

The displaced position of the CoPy changed forward with repetition of trials in both groups. In the initial set of trials, a significant difference was found between the first trial and the 12th trial in the young subjects, and between the first trial and 19th trial in the elderly subjects. This finding suggests that both the young and elderly are able to adapt dynamic postural control in response to backward postural disturbance, but the elderly subjects require more time for the adaptation. The displaced position was more forward in the second set of trials than in the initial set for both groups and more backward in the elderly than in the young for both sets. However, there was no significant group difference in the displaced position in the final phase of the adaptation (i.e., the mean of the last five trials in the second set). Thus, through 40 trials of repetition, the postural controllability in the elderly would reach almost the same level as in the young subjects.

We investigated age-related changes of frontal lobe function with postural adaptation, using late CNV. Late CNV reportedly reflects the motor preparation process and anticipatory attention directed to S2 [14,15]. Various cortical and subcortical regions, including the prefrontal cortex, supplementary motor area, premotor area, primary motor cortex, somatosensory cortex, and basal ganglia are involved in the production of late CNV [13]. In the present study, late CNV in the young started to increase negatively from the middle of the S1-S2 period and peaked just before S2. CNV peak amplitude was larger in the second set than in the initial set. These suggest that with the adaptation of the dynamic postural control, the young adults would direct a larger amount of attention to the disturbance, or make sufficient preparation for it, which would indicate frontal lobe activation during adaptation. In contrast to the young, late CNV in the elderly exhibited no negative increase as in the young, with no difference between the sets. This indicates that in spite of 40 trial repetitions, the elderly subjects were not able to direct enough attention and/or adequately prepare for the disturbance. In previous studies using a finger flexion task, CNV potentials in the elderly were reported to be small or positive [13,20]. These kinds of potentials are thought to be involved in the age-related degenerative processes, such as selective cortical aging and the decrease and degeneration of cortical synapses.

Late CNV in the elderly peaked at the middle of the S1-S2 period, which was followed by a gradual decrease toward S2. A slight increase of TA background activity started just before the CNV peak for both the young and elderly. It has been suggested that the decrease following the CNV peak would represent the onset of attentional shift to the objects other than S2, such as sensory information and output of motor command [11,12,16]. In addition, the CNV potential reportedly decreased when the S1-S2 task was performed under anxious or fearful conditions [21,22]. In the present study, the slight and continuous increase of TA background activity would represent the increase of muscle tension against the disturbance, indicating a strategy of postural preparation [12]. The results of CNV and TA increase suggest that the young could execute sufficient postural preparation, based on the accurate prediction of the disturbance timing, but the elderly were not able to perform it well, because of the fear of postural disturbance or some anxiety. This would be also associated with the age-related decreases in the anticipatory control and focusing function of attention, in which the frontal lobe plays a central role, based on the sensory integration [23].

Probably, as a result of the insufficient preparation for the postural disturbance, in the elderly, more time for the adaptation was required, and the peak amplitude and time of TA burst in response to the disturbance were smaller and later than in the young. In addition, although the peak time was shortened with the adaptation for both groups, the shortening was markedly smaller in the elderly than in the young.

For the elderly, in spite of the improvement of the dynamic postural controllability, no adaptational change in late CNV was recognized. It has been reported that even though the elderly can perform the task as automatically as the young, the activated brain area during a finger movement task was larger in the elderly than in the young adults [24]. Brain areas related to anticipatory postural control have been reported to be the cerebellum [25,26], as well as the premotor and supplementary motor cortices [27,28]. Therefore, it is possible that the elderly in the present study used various brain regions for dynamic postural control adaptation.

If the late adaptations in the elderly demonstrated in this study were caused not by the insufficiency but by the deterioration of the frontal lobe, then the adaptational change in the CNV would be found by more repetitions of postural disturbance. This question needs further investigation to be answered.

## Conclusions

Both groups showed better adaptation to the backward postural disturbance in the late set of trials than in the early set, but in the elderly the adaptation occurred later compared with young adults. In the process of adaptation, late CNV was markedly increased in the young adults but not in the elderly. It is conceivable that reduced activation of the frontal lobe may be one of the factors contributing to the delay in postural adaptability in the elderly. Elderly subjects may use various brain regions for the adaptation of dynamic postural control compared with young adults.

## Abbreviations

ANOVA: analysis of variance; BF: biceps femoris; CNV: contingent negative variation; CoPy: center of pressure in the anteroposterior direction; EBL: extreme backward leaning; EEG: electroencephalogram; EMG: electromyography; EOG: electrooculography; ES: erector spinae; FL: foot length; GcM: gastrocnemius; %FL: percentage distance from the heel in relation to FL; QS: quiet standing; RA: rectus abdominis; rEMG: full-wave-rectified EMG; RF: rectus femoris; S1: warning signal; S2: response signal; SD: standard deviation; Sol: soleus; TA: tibialis anterior.

## **Competing interests**

The authors declare that they have no competing interests.

## **Authors' contributions**

Contribution of each author is as follows: KF presented all the idea of this study, planed the method, directed the experiments and interpreted the results. Most sentences in Introduction and Discussion including Conclusion were written by KF. MM, NK and CY contributed to the experiments, data analyses and wrote the sections of Experimental procedures and Results. They also discussed about all part of manuscript with KF. All authors read and approved the final manuscript.
